# Synthesis, XRD Studies and NLO Properties of [*p*-H_2_NC_6_H_4_CH_2_NH_3_][B_5_O_6_(OH)_4_]·1/2H_2_O and NLO Properties of Some Related Pentaborate(1−) Salts

**DOI:** 10.1007/s10876-017-1205-1

**Published:** 2017-04-01

**Authors:** Michael A. Beckett, Simon J. Coles, Peter N. Horton, Charlotte L. Jones, Kerstin Krueger

**Affiliations:** 1 School of Chemistry, Bangor University, Bangor, LL57 2UW UK; 2 School of Chemistry, Southampton University, Southampton, SO17 IBJ UK

**Keywords:** Pentaborates, NLO properties, X-ray structure, SHG properties

## Abstract

The non-metal cation pentaborate(1−) salt [*p*-H_2_NC_6_H_4_CH_2_NH_3_][B_5_O_6_(OH)_4_]·1/2H_2_O (**1**) was synthesised from B(OH)_3_ and *p*-H_2_NC_6_H_4_CH_2_NH_2_ and crystallized from aqueous solution. Compound **1** was characterized by thermal (TGA/DSC), spectroscopic (IR, NMR) and single-crystal X-ray diffraction methods and it was found to crystallize in the non-centrosymmetric point group *P*2_1_. Powder SHG measurements on **1** and some related alkylammonium pentaborate salts, [NH_3_CMe_2_(CH_2_OH)][B_5_O_6_(OH)_4_], [NH_3_CMe(CH_2_OH)_2_][B_5_O_6_(OH)_4_] and [NH_3_CHMeCH_2_OH][B_5_O_6_(OH)_4_], a substituted imidazolium salt, [1,2,3-Me_3_C_3_H_2_N_2_][B_5_O_6_(OH)_4_], a substituted piperidinium salt, [(CH_2_)_5_NH(CH_2_CH_2_OH)][B_5_O_6_(OH)_4_], and a substituted pyrrolidinium salt, [*S*-(+)-2-(HOCH_2_)C_4_H_7_NH_2_][B_5_O_6_(OH)_4_], were determined. Compound **1** and all compounds, except [1,2,3-Me_3_C_3_H_2_N_2_][B_5_O_6_(OH)_4_], showed some weak SHG activity with SHG efficiencies of 0.1–0.2 relative to that of KH_2_PO_4_ (KDP).

**Electronic supplementary material:**

The online version of this article (doi:10.1007/s10876-017-1205-1) contains supplementary material, which is available to authorized users.

## Introduction

**Fig. 1:**
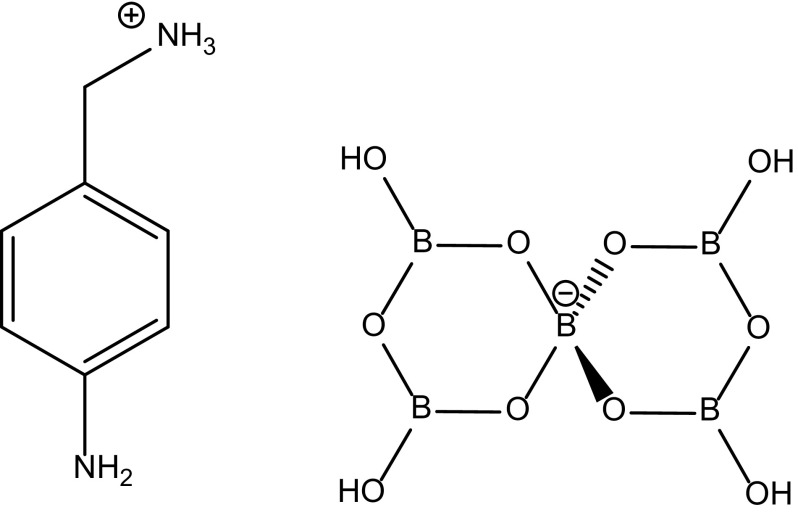
Schematic representation of the cation and anion present in [*p*-H_2_NC_6_H_4_CH_2_NH_3_][B_5_O_6_(OH)_4_]·1/2H_2_O (**1**)

Non-metal cation (NMC) pentaborate(1−) salts are readily prepared via cation templated self-assembly processes either by crystallization from aqueous solution [1–3] or by hydrothermal [4, 5] methods [6]. We have prepared a number of [NMC][B_5_O_6_(OH)_4_] salts by the former method [7–12]. The principal aim of our studies was to investigate the role of the cation in these templating reactions using structural (XRD) and computational (DFT) methods [13, 14]. During the course of these studies we recently prepared the salt [*p*-H_2_NC_6_H_4_CH_2_NH_3_][B_5_O_6_(OH)_4_)]·1/2H_2_O (**1**) (Fig. 1). Compound **1** has been characterized by XRD studies and it was found to crystallize in a non-centrosymmetric point group (*P*2_1_), a pre-requisite for non-linear optical (NLO) activity. The second-harmonic generation (SHG) properties of BBO (β-BaB_2_O_4_) and LBO (LiB_3_O_5_) are well-known [15] and the NLO properties of many borate crystals have now been investigated. Many of the structures investigated for SHG properties have been on metal containing borates [16] and relatively few have had non-metal cations as partners [17–20]. This manuscript describes the synthesis and characterization **1**, including it solid-state structure, and an investigation into its thermal and NLO (SHG) properties. A review of the structures of non-metal cation pentaborates that we had previously prepared [11–13] prompted us to re-synthesize those with non-centrosymmetric point groups in order to examine their NLO properties. This manuscript also reports on these results.

## Results and Discussion

### Synthesis and Characterization

**Fig. 2:**
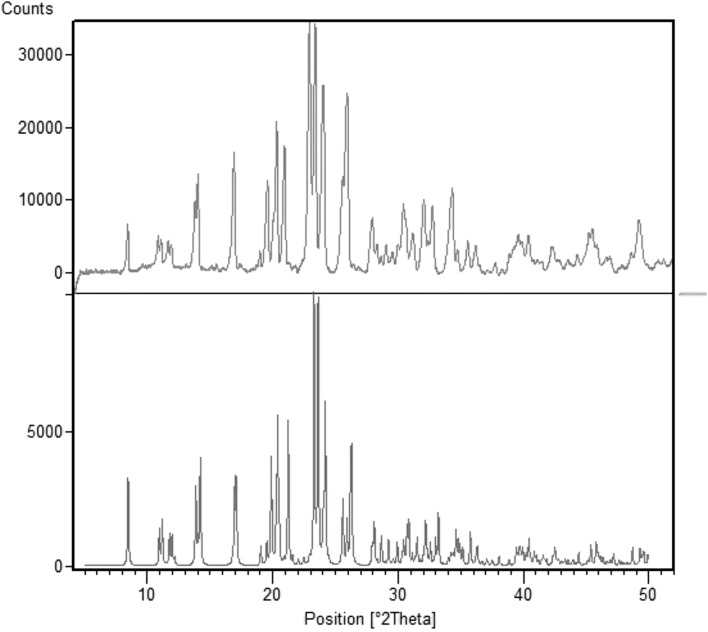
Powder-XRD patterns for **1**. Crude product (*top*) and simulated pattern (*bottom*) based on single-crystal XRD data

The salt [*p*-H_2_NC_6_H_4_CH_2_NH_3_][B_5_O_6_(OH)_4_)]·1/2H_2_O (**1**) was prepared in quantitative yield as a crystalline white/colourless solid from methanolic water (1:1) solution containing *p*-aminobenzylamine with boric acid (1:5). It was recrystallized from aqueous solution for single-crystal XRD studies. Compound **1** is formulated as a hemi-hydrate by XRD (“Solid-State Structure of 1” section), and thermal (“Thermal Properties” section) methods. Elemental analysis data are consistent with this formulation. Salt **1** was further characterized by spectroscopic (IR, NMR) analysis. NMR (^1^H, ^11^B, ^13^C) spectra for **1** were recorded in D_2_O solution and ^1^H and ^13^C spectra showed the expected signals. The ^1^H spectrum had equal intensity aromatic signals at 6.85 and 7.24 ppm (both AB doublets) and a further equal intensity aliphatic signal at 4.04 ppm. The BOH and NH protons were rapidly exchanging in D_2_O and combined with the HOD signal at 4.79 ppm. Five signals were observed in the ^13^C (DEPTQ) spectrum, with four in the aromatic region and one at 42.8 ppm. In accord with ^11^B spectra of previously reported compounds [7–12] the ^11^B spectrum of **1** gave three signals at +1.2, +13.0, +18.1 ppm consistent with equilibria linking monoborate and polyborate anions [21, 22]. A ‘dilute’ ^11^B spectrum (D_2_O) shows only one signal at +15.7 which is in agreement with a boron/charge ratio of 5 [13]. The IR spectrum of **1** showed strong B–O bands [23] and the diagnostic pentaborate(1−) band at 919 cm^−1^ was present [10]. A powder XRD spectrum of the crude product (Fig. 2) demonstrated that the crude product was crystalline and since it had a pattern matching that predicted from the single-crystal XRD analysis, confirmed that the single-crystals were representative of the crude product. Powder SHG measurements (“SHG Properties of 1 and Some Related Pentaborate(1−) Salts” section) were performed on the crude sample.

### Solid-State Structure of 1

**Fig. 3:**
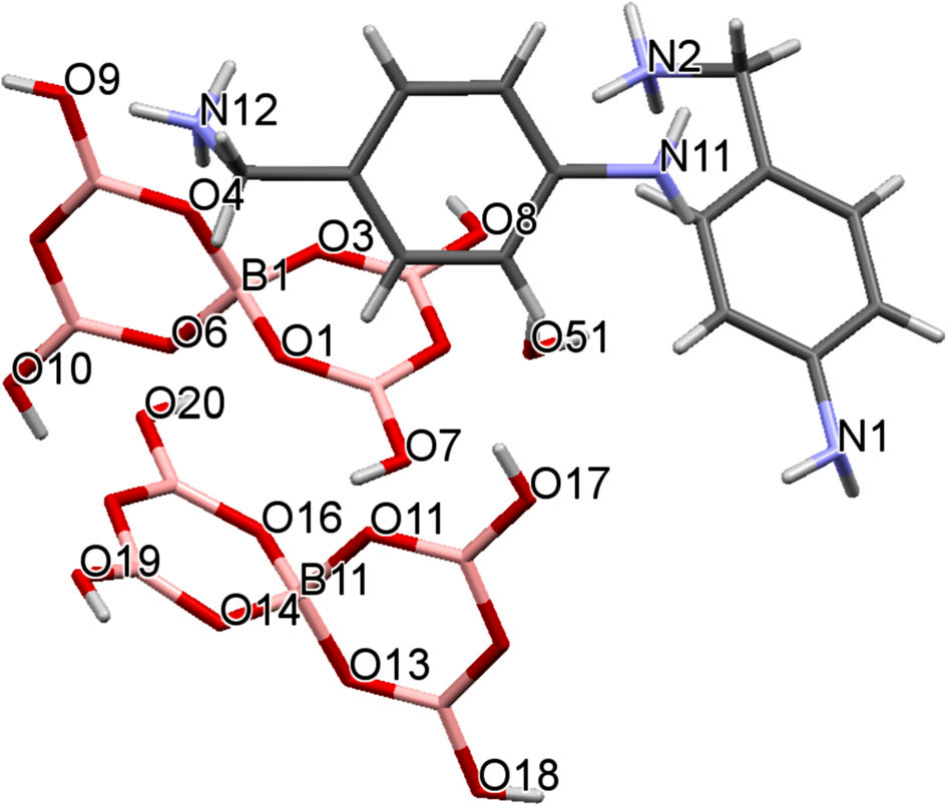
Drawing **1** showing (selected) numbering scheme. B_tet_–O bond lengths range from 1.452(3) to 1.488(3) Å, av. 1.473 Å; B_trig_–O bond lengths range from 1.349(3) to 1.391(3) Å, av. 1.370 Å; O–B_tet_–O angles range from 106.57(15)° to 110.67(16)°, av. 109.47°; O–B_trig_–O angles range from 115.72(18)° to 122.96(19)°, av. 119.99°; B–O–B angles range from 117.60(17) to 124.11(17), av. 121.46°

**Fig. 4:**
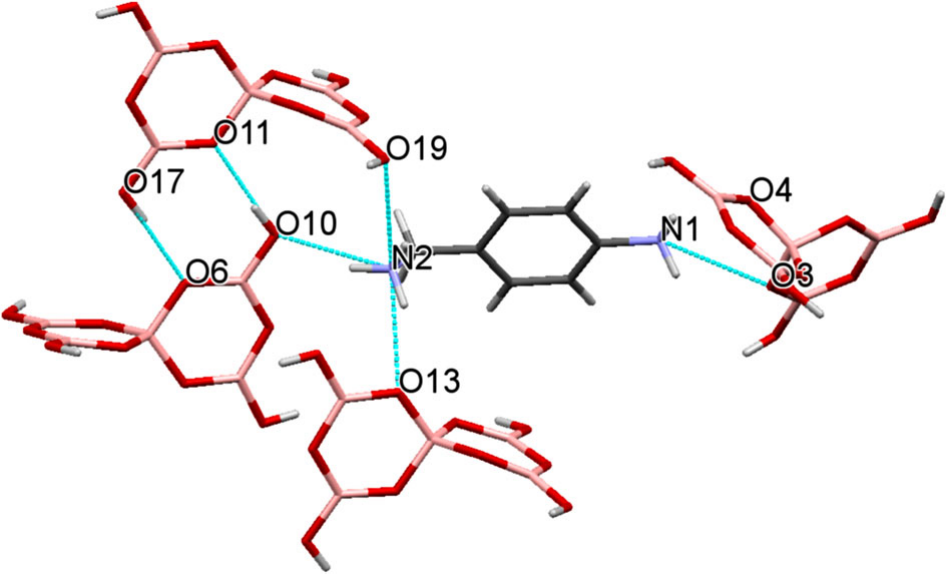
H-bond donor interactions (*blue*) originating from the cation containing N1. The 5 interactions are to 3 α (to O3, O4 and O13) and 2 β (to O10 and O19) sites of the adjacent pentaborate(1−) anions. The interaction N1–H1B…O4 is slightly longer and not shown in *blue*. An R_2_^2^(8) interaction between two pentaborate(1−) anions and involving O10H10…O11 and O17H17…O6 is also shown (Color figure online)

Crystal data and structure refinement data for compound **1** is given in Table 1. Crystals are monoclinic with a space group of *P*2_1_. The formula unit has two independent *p*-aminobenzylammonium cations, two pentaborate(1−) anions and one water of crystallization, with an effective *Z* of 2 (Fig. 3). Many non-metal cation pentaborate salts have structures which can be interpreted as based solely on giant H-bonded anion–anion cages with cations and interstitial molecules located within the cavities [1, 2, 13]. This is not the case for **1** where the water molecules link between pentaborate(1−) anions and form part of the giant network (rather than as a cavity filler) resulting in a unique structure. Bond lengths and bond angles within the boroxole rings of the pentaborates are within ranges commonly seen in other pentaborate(1−) salts [4, 5, 7–13, 24–26] and in related boroxole containing systems [27–29]. Some summary data are given in the legend to Fig. 2, and full details can be found in the supplementary material. Each pentaborate(1−) forms four donor H-bonds to three neighbouring pentaborate(1−) anions (two at α-sites and one at a β site [3]) and one amino nitrogen of the cation. The α-interactions with neighbours are paired e.g. O10H10···O11 and O17H17···O6 resulting in energetically favourable [13, 30] eight membered rings, R_2_^2^(8) [31] (Fig. 4). The H_2_O molecule (containing O51) is involved in donor H-bonds to O8 (β site) and O14 (α site) bridging two pentaborate(1−) anions and O51 also accepts two H-bonds from N11H1A and N12H12C. All amino hydrogen atoms in the two cations are involved in H-bond donor interactions: the cation containing N1 forms five donor H-bonds to three α and two β pentaborate(1−) sites whilst the cation containing N11 forms five donor H-bonds to two water molecules, two β and one α pentaborate(1−) site. The donor H-bond interactions from the cation containing N1 are shown in Fig. 4. Full details of H-bonding interactions are in the supplementary material.

**Table 1:** Crystal data and structure refinement

Empirical formula	C_7_H_16_B_5_N_2_O_10.50_
Formula weight	350.27
Temperature	100(2) K
Wavelength	0.71075 Å
Crystal system	Monoclinic
Space group	*P*2_1_
Unit cell dimensions	*a* = 8.9556(5) Å, *α* = 90°
	*b* = 20.8702(15) Å, *β* = 118.0750(10)°
	*c* = 9.1414(9) Å, *γ* = 90°
Volume	1507.5(2) Å^3^
*Z*	4
Density (calculated)	1.543 Mg/m^3^
Absorption coefficient	0.134 mm^**−1**^
*F(000)*	724
Crystal	Blade; colourless
Crystal size	0.210 × 0.110 × 0.030 mm^3^
*θ* range for data collection	3.192°–27.478°
Index ranges	−11 ≤ *h* ≤ 11, −27 ≤ *k* ≤ 27, −11 ≤ *l* ≤ 11
Reflections collected	18,862
Independent reflections	6843 [*R*_*int*_ = 0.0415]
Completeness to *θ* = 25.242°	99.6%
Absorption correction	Semi-empirical from equivalents
Max. and min. transmission	1.000 and 0.801
Refinement method	Full-matrix least-squares on *F* ^2^
Data/restraints/parameters	6843/20/498
Goodness-of-fit on *F* ^2^	1.046
Final *R* indices [*F* ^2^ > 2*σ*(*F* ^2^)]	*R*1 = 0.0301, *wR2* = 0.0762
*R* indices (all data)	*R*1 = 0.0323, *wR2* = 0.0775
Absolute structure parameter	0.3(3)
Largest diff. peak and hole	0.204 and −0.183 e Å^**−3**^

### Thermal Properties

The thermal properties of **1** were investigated by TGA/DSC analysis. Compound **1** is thermally decomposed in air (to 2.5 B_2_O_3_) via a multi-stage process. The water of crystallization is lost at ~120 °C (–0.5H_2_O) and further dehydration (pentaborate cross linking, 2BOH → B–O–B + H_2_O) occurs at ~200 °C (–2H_2_O), with the formation of an anhydrous pentaborate, [H_2_NC_6_H_4_CH_2_NH_3_][B_5_O_8_]. These processes are endothermic. At higher temperatures (200–800 °C) oxidation of the organic cation occurs (exothermic) and this results in the formation of a boron oxide residue. These observations are consistent with the decomposition of other non-metal pentaborate(1−) compounds in air [10–13] and under an inert (Ar/N_2_) atmosphere [1, 2, 32, 33].

### SHG Properties of 1 and Some Related Pentaborate(1−) Salts

Compound **1** crystallized in a non-centrosymmetric point group and this prompted us to investigate its NLO (SHG) properties. Powdered crystals of **1** were examined using the Kurtz powder [34] method with a YAG:Nd^3+^ laser (λ = 1064 nm) as the source and frequency doubled outputs (λ = 532 nm) results were compared with those obtained from KDP (KH_2_PO_4_) which used as a reference. Compound **1** displayed NLO properties but its SHG efficiency was only 0.1 of that of the KDP standard. Inspection of XRD data of a number of other non-metal cation pentaborate salts which had previously been synthesized in our laboratories revealed that some of them also crystallized in non-centrosymmetric points groups. These compounds [11–13] were re-synthesized and their SHG efficiencies were also determined. The alkylammonium pentaborate salts [NH_3_CMe_2_(CH_2_OH)][B_5_O_6_(OH)_4_] [11], [NH_3_CMe(CH_2_OH)_2_][B_5_O_6_(OH)_4_] [11] and [NH_3_CHMeCH_2_OH][B_5_O_6_(OH)_4_] [11] all had SHG efficiencies of 0.2. The substituted imidazolium salt, [1,2,3-Me_3_C_3_H_2_N_2_][B_5_O_6_(OH)_4_] [12], had no measureable SHG activity, whilst the substituted piperidinium salt [(CH_2_)_5_NH(CH_2_CH_2_OH)][B_5_O_6_(OH)_4_] [11] and the substituted pyrrolidinium salt [*S*-(+)-2-(HOCH_2_)C_4_H_7_NH_2_][B_5_O_6_(OH)_4_] [13] had SHG efficiencies of 0.1 and 0.2, respectively. As a further control we synthesised crystals of the literature compound [N(CH_2_CH_2_)_3_NH][B_5_O_6_(OH)_4_] [19] and obtained a SHG efficiency of 0.6. This value was lower than that reported (0.9) but nevertheless it was significantly higher than that of the other pentaborate(1−) salts that we describe above.

## Experimental

### General

All chemicals were obtained commercially. NMR spectra were obtained on a Bruker Avance-400 spectrometer with ^1^H ^11^B, ^13^C spectra (D_2_O) obtained at 400, 128, and 101 MHz, respectively. Fourier transform Infrared spectra (FTIR) were obtained as KBr pellets on a Perkin-Elmer 100 FTIR spectrometer over 450–4000 cm^−1^. TGA and DSC analysis was performed between 10 and 800 °C (in air) on an SDT Q600 V4.1 Build 59 instrument using Al_2_O_3_ crucibles, with a ramp temperature rate of 10 °C min^−1^. Powder XRD were obtained on a Philips 1050/37 X-ray diffractometer equipped with an iron filter, using Cu-Kα radiation (λ = 0.154056 nm) with a continuous scan between 2θ = 5°–75° and Philips E’Pert software. Single-crystal X-ray crystallography was carried out at the EPSRC National Crystallography service at the University of Southampton. SHG measurements were obtained by Prof. P. Das, Bangalore, India. CHN analysis was carried out at OEA laboratories Ltd in Callington, Cornwall. With the exception of **1** (see below) samples used in the powder SHG measurements were all prepared by literature methods [11–13, 19] and had characterization data consistent with published data.

### Synthesis, Spectroscopic and Analytical Data for 1

B(OH)_3_ (5.01 g, 81 mmol) was dissolved in 1:1 MeOH:H_2_O (100 ml). 4-Aminobenzylamine (1.98 g, 16.2 mmol) was added with stirring. After 1 h the solvent was removed to yield the product as a white solid, which was oven dried at 60 °C for 24 h (5.46 g, 99%). Recrystallization of a portion of the crude product from H_2_O yielded colourless crystals of **1** which were suitable for single-crystal XRD analysis. Elemental analysis (%) for **1**, C_7_H_16_N_2_B_5_O_10.5_. Calc. (%): C, 24.0; H, 4.6, N, 8.0; Crude, Found (%): C, 24.5; H, 4.7; N, 8.0, Recrystallized, Found (%): C, 24.0; H, 4.6; N, 7.7. ^1^H NMR (400 MHz, D_2_O): 4.04 (s, 2H, C*H*_2_), 4.79 (s, *H*OD, O*H* and N*H* rapidly exchanging in the D_2_O), 6.85 (d, 2*H*, arom., ^3^ *J* = 8.0 Hz), 7.24 (d, 2*H*, arom, ^3^ *J* = 8.0 Hz). ^13^C NMR (101 MHz, D_2_O): 42.77 (*C*H_2_), 116.47 (2*C*H), 123.18 (*C*CH_2_), 130.19 (2*C*H), 147.06 (*C*NH_2_). ^11^B NMR(128 MHz, D_2_O): 1.2, 13.0, 18.1. IR (KBr pellets, ν_max_/cm^−1^): 3487 (m), 3171 (br,s), 1621 (m), 1526 (m), 1428 (br,m), 1377 (s), 1327 (s), 1190 (s), 1059 (s), 1018 (s), 919 (vs), 842 (s), 775 (s), 699 (s), 552 (m), 472 (m). p-XRD {d-spacing/A (% rel. int.)}: 3.88 (100), 3.80 (97), 3.70 (71), 3.43 (70), 4.37 (57), 4.24 (48). TGA: Loss of 0.5H_2_O (110–120 °C): 2.6% (2.6% calc); loss of 2H_2_O (180–200 °C): 12.2% (12.9% calc.); oxidation of organics (200–800 °C to leave 2.5B_2_O_3_): residue 51.3% (49.7% calc.).

### X-ray Crystallography

A suitable crystal of compound **1** was selected and measured following a standard method [35] on a Rigaku AFC12 goniometer equipped with a an enhanced sensitivity (HG) Saturn724+ detector mounted at the window of an FR-E+ SuperBright molybdenum rotating anode with HF Varimax optics (100 µm focus) at 100(2) K. Cell determination, data collection, data reduction, cell refinement and absorption correction was carried out using CrystalClear-SM Expert 3.1 b27 (Rigaku 2012) [36]. The structure was solved using SUPERFLIP [37] and refined using SHELXL-2013 [38].

## Conclusion

In summary, an new isolated non-metal cation pentaborate(1−) salt, [*p*-H_2_NC_6_H_4_CH_2_NH_3_][B_5_O_6_(OH)_4_]·1/2H_2_O, was synthesised from boric acid and the free amine by crystallization from a methanolic aqueous solution. The title compound crystallizes in a non-centrosymmetric point group and shows a weak SHG effect (SHG efficiency of 0.2 of KDP) using the Kurtz powder method. The SHG efficiency is of a similar magnitude to that of other non-metal pentaborate(1−) salts which we have previously prepared and which also crystallize in non-centrosymmetric point groups.

## Associated Content

CCDC 1534385 contains the supplementary crystallographic data for this paper. These data can be obtained free of charge from The Cambridge Crystallographic Data Centre via www.ccdc.cam.ac.uk/data_request/cif. Crystallographic data are also available as supplementary material.

## Electronic supplementary material

Below is the link to the electronic supplementary material.

Supplementary material 1 (DOCX 259 kb)

Supplementary material 2 (PDF 1380 kb)

Supplementary material 3 (PDF 790 kb)

Supplementary material 4 (PDF 118 kb)
