# Supplementary material for: Synthesis, XRD Studies and NLO Properties of [p-H2NC6H4CH2NH3][B5O6(OH)4]·1/2H2O and NLO Properties of Some Related Pentaborate(1−) Salts
Source: J Clust Sci. 2017 Apr 1;28(4):2087–95. doi: 10.1007/s10876-017-1205-1 (PMC7098061; doi:10.1007/s10876-017-1205-1)

| 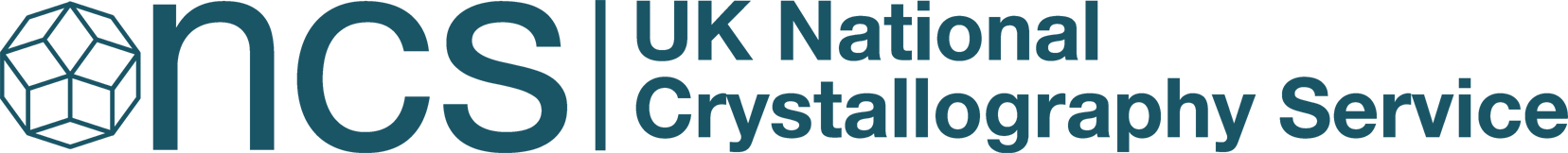 |
| --- |

**SUPPLEMENTARY CRYSTALLOGRAPHIC DATA**

**Synthesis, XRD studies and NLO properties of [*p*-H_2_NC_6_H_4_CH_2_NH_3_][B_5_O_6_(OH)_4_]^.^1/2H_2_O and NLO properties of some related pentaborate(1-) salts.** Michael A. Beckett,* Peter N. Horton, Simon J. Coles, Charlotte L. Jones, and Kerstin Krueger.

**Table 1.** Crystal data and structure refinement.

|  |
| --- |

Identification code **2014ncs0055 / CLJ/MAB/1/156R**

Empirical formula C_7_H_16_B_5_N_2_O_10.50_

Formula weight 350.27

Temperature 100(2) K

Wavelength 0.71075 Å

Crystal system Monoclinic

Space group *P*2_1_

Unit cell dimensions *a* = 8.9556(5) Å *α* = 90°

*b* = 20.8702(15) Å *β* = 118.0750(10)°

*c* = 9.1414(9) Å *γ* = 90°

Volume 1507.5(2) Å^3^

*Z* 4

Density (calculated) 1.543 Mg / m^3^

Absorption coefficient 0.134 mm**^−1^**

*F(000)* 724

Crystal Blade; Colourless

Crystal size 0.210 × 0.110 × 0.030 mm^3^

*θ* range for data collection 3.192 − 27.478°

Index ranges −11 ≤ *h* ≤ 11, −27 ≤ *k* ≤ 27, −11 ≤ *l* ≤ 11

Reflections collected 18862

Independent reflections 6843 [*R_int_* = 0.0415]

Completeness to *θ* = 25.242° 99.6 %

Absorption correction Semi−empirical from equivalents

Max. and min. transmission 1.000 and 0.801

Refinement method Full-matrix least-squares on *F*^2^

Data / restraints / parameters 6843 / 20 / 498

Goodness-of-fit on *F*^2^ 1.046

Final *R* indices [*F*^2^ > 2*σ*(*F*^2^)] *R1* = 0.0301, *wR2* = 0.0762

*R* indices (all data) *R1* = 0.0323, *wR2* = 0.0775

Absolute structure parameter 0.3(3)

Extinction coefficient n/a

Largest diff. peak and hole 0.204 and −0.183 e Å**^−3^**

|  |
| --- |

**Diffractometer:** *Rigaku AFC12* goniometer equipped with an enhanced sensitivity (HG) *Saturn724+* detector mounted at the window of an *FR-E+ SuperBright* molybdenum rotating anode generator with HF *Varimax* optics (100µm focus). **Cell determination and** **data collection**: *CrystalClear-SM Expert 3.1 b27* (Rigaku, 2013). **Data reduction, cell** **refinement and** **absorption correction**: *CrystalClear-SM Expert 3.1 b27* (Rigaku, 2012). S**tructure solution**:  *SUPERFLIP* (Palatinus, L. & Chapuis, G. (2007). J. Appl. Cryst. 40, 786-790.) S**tructure refinement**: *SHELXL-2013* (G Sheldrick, G.M. (2008). Acta Cryst. A64, 112-122.). **Graphics:** *ORTEP3 for Windows* (L. J. Farrugia, J. Appl. Crystallogr. 1997, 30, 565

**Special details**:

**Table 2.** Atomic coordinates [× 10^4^], equivalent isotropic displacement parameters [Å^2^ × 10^3^] and site occupancy factors. *U_eq_* is defined as one third of the trace of the orthogonalized *U^ij^* tensor.

Atom *x* *y* *z* *U_eq_* *S.o.f.*

B1 1143(3) 7524(1) 587(3) 13(1) 1

B2 4127(3) 7556(1) 2875(3) 16(1) 1

B3 2454(3) 6641(1) 2597(3) 16(1) 1

B4 −853(3) 7663(1) −2426(3) 14(1) 1

B5 −1384(3) 8243(1) −483(3) 15(1) 1

O1 2860(2) 7807(1) 1459(2) 15(1) 1

O2 3979(2) 6965(1) 3463(2) 20(1) 1

O3 1115(2) 6903(1) 1287(2) 16(1) 1

O4 662(2) 7457(1) −1183(2) 14(1) 1

O5 −1872(2) 8077(1) −2108(2) 16(1) 1

O6 −18(2) 7971(1) 800(2) 15(1) 1

O7 5598(2) 7890(1) 3764(2) 20(1) 1

O8 2337(2) 6040(1) 3153(2) 19(1) 1

O9 −1318(2) 7455(1) −3990(2) 17(1) 1

O10 −2320(2) 8707(1) −242(2) 19(1) 1

B11 7411(3) 9334(1) 3212(3) 14(1) 1

B12 9933(3) 8604(1) 4250(3) 15(1) 1

B13 9488(3) 9218(1) 6203(3) 14(1) 1

B14 6268(3) 10038(1) 749(3) 15(1) 1

B15 4496(3) 9213(1) 880(3) 15(1) 1

O11 8523(2) 8860(1) 2983(2) 15(1) 1

O12 10470(2) 8789(1) 5874(2) 16(1) 1

O13 7997(2) 9452(1) 4982(2) 14(1) 1

O14 7409(2) 9929(1) 2365(2) 15(1) 1

O15 4758(2) 9696(1) 0(2) 17(1) 1

O16 5686(2) 9050(1) 2450(2) 14(1) 1

O17 10888(2) 8143(1) 4023(2) 18(1) 1

O18 10002(2) 9407(1) 7798(2) 17(1) 1

O19 6549(2) 10465(1) −217(2) 19(1) 1

O20 2989(2) 8895(1) 79(2) 19(1) 1

C1 8994(3) 5578(1) 7369(3) 16(1) 1

C2 8026(3) 5790(1) 5749(3) 19(1) 1

C3 6853(3) 5385(1) 4556(3) 20(1) 1

C4 6606(3) 4766(1) 4980(3) 16(1) 1

C5 7563(3) 4558(1) 6606(3) 17(1) 1

C6 8768(3) 4959(1) 7796(3) 18(1) 1

C7 5331(3) 4329(1) 3682(3) 19(1) 1

N1 10230(2) 5983(1) 8589(2) 21(1) 1

N2 3601(2) 4622(1) 2925(2) 19(1) 1

C11 7703(3) 6231(1) 1134(3) 17(1) 1

C12 7384(3) 6815(1) 1672(3) 19(1) 1

C13 6274(3) 7253(1) 521(3) 19(1) 1

C14 5506(3) 7121(1) −1167(3) 18(1) 1

C15 5844(3) 6539(1) −1698(3) 19(1) 1

C16 6905(3) 6094(1) −567(3) 19(1) 1

C17 4290(3) 7586(1) −2423(3) 21(1) 1

N11 8913(2) 5799(1) 2248(2) 21(1) 1

N12 2494(2) 7372(1) −3043(2) 18(1) 1

O51 1762(2) 6094(1) 5909(2) 22(1) 1

|  |
| --- |

**Table 3.** Bond lengths [Å] and angles [°].

|  |
| --- |

B1−O3 1.452(3)

B1−O4 1.472(3)

B1−O6 1.476(3)

B1−O1 1.481(3)

B2−O1 1.362(3)

B2−O7 1.368(3)

B2−O2 1.377(3)

B3−O3 1.349(3)

B3−O8 1.376(3)

B3−O2 1.390(3)

B4−O9 1.358(3)

B4−O4 1.366(3)

B4−O5 1.383(3)

B5−O6 1.358(3)

B5−O10 1.365(3)

B5−O5 1.381(3)

O7−H7 0.8400

O8−H8 0.8400

O9−H9 0.8400

O10−H10 0.8400

B11−O14 1.462(3)

B11−O13 1.467(2)

B11−O16 1.487(3)

B11−O11 1.488(3)

B12−O11 1.358(3)

B12−O17 1.366(3)

B12−O12 1.382(2)

B13−O18 1.365(3)

B13−O13 1.365(3)

B13−O12 1.383(3)

B14−O19 1.359(3)

B14−O14 1.363(3)

B14−O15 1.391(3)

B15−O20 1.367(3)

B15−O16 1.368(3)

B15−O15 1.377(3)

O17−H17 0.8400

O18−H18 0.8400

O19−H19 0.8400

O20−H20 0.8400

C1−C2 1.390(3)

C1−C6 1.393(3)

C1−N1 1.422(3)

C2−C3 1.389(3)

C2−H2 0.9500

C3−C4 1.396(3)

C3−H3 0.9500

C4−C5 1.391(3)

C4−C7 1.506(3)

C5−C6 1.393(3)

C5−H5 0.9500

C6−H6 0.9500

C7−N2 1.497(3)

C7−H7A 0.9900

C7−H7B 0.9900

N1−H1A 0.873(14)

N1−H1B 0.885(14)

N2−H2A 0.890(13)

N2−H2B 0.894(13)

N2−H2C 0.895(13)

C11−C12 1.392(3)

C11−C16 1.401(3)

C11−N11 1.410(3)

C12−C13 1.395(3)

C12−H12 0.9500

C13−C14 1.389(3)

C13−H13 0.9500

C14−C15 1.393(3)

C14−C17 1.507(3)

C15−C16 1.382(3)

C15−H15 0.9500

C16−H16 0.9500

C17−N12 1.500(3)

C17−H17A 0.9900

C17−H17B 0.9900

N11−H11A 0.875(14)

N11−H11B 0.889(14)

N12−H12A 0.884(13)

N12−H12B 0.893(13)

N12−H12C 0.904(13)

O51−H51A 0.861(13)

O51−H51B 0.866(13)

O3−B1−O4 110.01(16)

O3−B1−O6 110.26(16)

O4−B1−O6 110.25(16)

O3−B1−O1 110.32(16)

O4−B1−O1 108.83(16)

O6−B1−O1 107.12(16)

O1−B2−O7 120.53(19)

O1−B2−O2 121.66(19)

O7−B2−O2 117.81(19)

O3−B3−O8 120.4(2)

O3−B3−O2 122.22(18)

O8−B3−O2 117.31(19)

O9−B4−O4 117.84(18)

O9−B4−O5 121.00(18)

O4−B4−O5 121.15(18)

O6−B5−O10 121.94(19)

O6−B5−O5 121.67(19)

O10−B5−O5 116.36(18)

B2−O1−B1 123.27(16)

B2−O2−B3 117.60(17)

B3−O3−B1 124.11(17)

B4−O4−B1 123.09(17)

B5−O5−B4 118.47(17)

B5−O6−B1 123.62(17)

B2−O7−H7 109.5

B3−O8−H8 109.5

B4−O9−H9 109.5

B5−O10−H10 109.5

O14−B11−O13 110.67(16)

O14−B11−O16 110.41(16)

O13−B11−O16 109.66(16)

O14−B11−O11 108.95(17)

O13−B11−O11 110.49(16)

O16−B11−O11 106.57(15)

O11−B12−O17 122.96(19)

O11−B12−O12 121.31(18)

O17−B12−O12 115.72(18)

O18−B13−O13 118.60(19)

O18−B13−O12 119.35(18)

O13−B13−O12 122.04(18)

O19−B14−O14 122.7(2)

O19−B14−O15 116.79(19)

O14−B14−O15 120.49(19)

O20−B15−O16 121.77(19)

O20−B15−O15 116.07(18)

O16−B15−O15 122.14(19)

B12−O11−B11 123.85(16)

B12−O12−B13 118.61(16)

B13−O13−B11 123.14(17)

B14−O14−B11 121.92(17)

B15−O15−B14 118.77(17)

B15−O16−B11 121.13(16)

B12−O17−H17 109.5

B13−O18−H18 109.5

B14−O19−H19 109.5

B15−O20−H20 109.5

C2−C1−C6 119.59(19)

C2−C1−N1 120.80(19)

C6−C1−N1 119.60(19)

C3−C2−C1 120.3(2)

C3−C2−H2 119.8

C1−C2−H2 119.8

C2−C3−C4 120.4(2)

C2−C3−H3 119.8

C4−C3−H3 119.8

C5−C4−C3 119.14(19)

C5−C4−C7 120.70(18)

C3−C4−C7 120.16(18)

C4−C5−C6 120.57(19)

C4−C5−H5 119.7

C6−C5−H5 119.7

C1−C6−C5 119.96(19)

C1−C6−H6 120.0

C5−C6−H6 120.0

N2−C7−C4 110.69(17)

N2−C7−H7A 109.5

C4−C7−H7A 109.5

N2−C7−H7B 109.5

C4−C7−H7B 109.5

H7A−C7−H7B 108.1

C1−N1−H1A 112(2)

C1−N1−H1B 110.8(19)

H1A−N1−H1B 109(3)

C7−N2−H2A 106.9(19)

C7−N2−H2B 110.8(17)

H2A−N2−H2B 110.0(16)

C7−N2−H2C 110.2(18)

H2A−N2−H2C 110.0(16)

H2B−N2−H2C 109.1(16)

C12−C11−C16 119.20(19)

C12−C11−N11 121.38(19)

C16−C11−N11 119.21(19)

C11−C12−C13 119.91(19)

C11−C12−H12 120.0

C13−C12−H12 120.0

C14−C13−C12 120.9(2)

C14−C13−H13 119.6

C12−C13−H13 119.6

C13−C14−C15 118.96(19)

C13−C14−C17 121.4(2)

C15−C14−C17 119.59(19)

C16−C15−C14 120.7(2)

C16−C15−H15 119.7

C14−C15−H15 119.7

C15−C16−C11 120.3(2)

C15−C16−H16 119.8

C11−C16−H16 119.8

N12−C17−C14 110.98(17)

N12−C17−H17A 109.4

C14−C17−H17A 109.4

N12−C17−H17B 109.4

C14−C17−H17B 109.4

H17A−C17−H17B 108.0

C11−N11−H11A 116(2)

C11−N11−H11B 113(2)

H11A−N11−H11B 111(3)

C17−N12−H12A 112.7(17)

C17−N12−H12B 110.7(19)

H12A−N12−H12B 108.7(16)

C17−N12−H12C 111.0(16)

H12A−N12−H12C 107.2(16)

H12B−N12−H12C 106.4(16)

H51A−O51−H51B 104.2(18)

|  |
| --- |

Symmetry transformations used to generate equivalent atoms:

|  |
| --- |

**Table 4.** Anisotropic displacement parameters [Å^2^× 10^3^]. The anisotropic displacement

factor exponent takes the form: −2*π* ^2^[*h*^2^*a**^2^*U*^11^ + ... + 2 *h k a** *b** *U*^12^ ].

Atom *U*^11^ *U*^22^ *U*^33^ *U*^23^ *U*^13^ *U*^12^

B1 13(1) 12(1) 13(1) 0(1) 5(1) 1(1)

B2 16(1) 16(1) 14(1) 2(1) 6(1) 2(1)

B3 18(1) 17(1) 15(1) 0(1) 9(1) 2(1)

B4 14(1) 14(1) 14(1) 2(1) 6(1) 0(1)

B5 16(1) 14(1) 14(1) 1(1) 6(1) 0(1)

O1 14(1) 15(1) 14(1) 2(1) 4(1) 0(1)

O2 16(1) 19(1) 18(1) 6(1) 2(1) −1(1)

O3 15(1) 14(1) 15(1) 1(1) 5(1) −1(1)

O4 14(1) 16(1) 12(1) 1(1) 5(1) 3(1)

O5 16(1) 18(1) 13(1) 0(1) 5(1) 4(1)

O6 15(1) 15(1) 12(1) 0(1) 5(1) 2(1)

O7 16(1) 18(1) 17(1) 4(1) 1(1) −1(1)

O8 19(1) 18(1) 20(1) 4(1) 7(1) 0(1)

O9 15(1) 21(1) 12(1) −1(1) 4(1) 3(1)

O10 21(1) 21(1) 12(1) 1(1) 6(1) 7(1)

B11 14(1) 15(1) 12(1) 0(1) 6(1) 1(1)

B12 15(1) 16(1) 13(1) 1(1) 6(1) 1(1)

B13 14(1) 13(1) 15(1) 0(1) 6(1) −1(1)

B14 16(1) 15(1) 15(1) 2(1) 8(1) 2(1)

B15 15(1) 16(1) 14(1) 0(1) 6(1) 1(1)

O11 16(1) 16(1) 12(1) 0(1) 6(1) 3(1)

O12 16(1) 19(1) 12(1) 0(1) 5(1) 4(1)

O13 15(1) 16(1) 10(1) 0(1) 5(1) 2(1)

O14 15(1) 14(1) 14(1) 1(1) 5(1) 0(1)

O15 16(1) 18(1) 13(1) 2(1) 4(1) −1(1)

O16 14(1) 16(1) 12(1) 1(1) 5(1) −1(1)

O17 21(1) 21(1) 12(1) 1(1) 7(1) 7(1)

O18 15(1) 19(1) 12(1) −1(1) 3(1) 3(1)

O19 18(1) 20(1) 15(1) 3(1) 6(1) −2(1)

O20 16(1) 19(1) 15(1) 5(1) 2(1) −2(1)

C1 12(1) 19(1) 18(1) −5(1) 7(1) −1(1)

C2 18(1) 17(1) 23(1) 2(1) 10(1) −1(1)

C3 17(1) 22(1) 17(1) 2(1) 6(1) −2(1)

C4 15(1) 18(1) 16(1) −3(1) 8(1) −1(1)

C5 19(1) 16(1) 18(1) 0(1) 10(1) 1(1)

C6 17(1) 20(1) 16(1) 1(1) 8(1) 2(1)

C7 18(1) 18(1) 19(1) −4(1) 8(1) −3(1)

N1 20(1) 20(1) 21(1) −7(1) 8(1) −1(1)

N2 18(1) 20(1) 16(1) −2(1) 7(1) −4(1)

C11 13(1) 18(1) 20(1) 3(1) 8(1) 0(1)

C12 19(1) 20(1) 18(1) 0(1) 8(1) −1(1)

C13 19(1) 18(1) 22(1) 0(1) 11(1) 2(1)

C14 13(1) 21(1) 20(1) 3(1) 8(1) 1(1)

C15 14(1) 26(1) 17(1) −1(1) 7(1) −2(1)

C16 17(1) 19(1) 23(1) −3(1) 12(1) −1(1)

C17 18(1) 22(1) 24(1) 6(1) 9(1) 1(1)

N11 20(1) 18(1) 22(1) 3(1) 8(1) 3(1)

N12 18(1) 19(1) 18(1) 3(1) 9(1) 4(1)

O51 26(1) 19(1) 22(1) 4(1) 12(1) 3(1)

|  |
| --- |

**Table 5.** Hydrogen coordinates [× 10^4^] and isotropic displacement parameters [Å^2^ × 10^3^].

Atom *x* *y* *z* *U_eq_* *S.o.f.*

H7 5549 8240 3289 29 1

H8 1318 5925 2702 29 1

H9 −2301 7584 −4631 25 1

H10 −1936 8772 779 28 1

H17 10513 8077 3004 27 1

H18 10909 9220 8432 25 1

H19 7523 10621 320 28 1

H20 3033 8557 598 29 1

H2 8167 6214 5456 23 1

H3 6215 5530 3446 23 1

H5 7393 4140 6909 20 1

H6 9435 4809 8898 21 1

H7A 5663 4249 2806 23 1

H7B 5314 3913 4193 23 1

H1A 10090(40) 6014(16) 9470(30) 41(9) 1

H1B 10190(40) 6372(8) 8190(30) 30(8) 1

H2A 3310(30) 4653(12) 3730(30) 35(8) 1

H2B 3610(30) 5010(7) 2520(30) 26(7) 1

H2C 2860(30) 4372(10) 2110(20) 29(7) 1

H12 7921 6914 2821 23 1

H13 6040 7646 897 23 1

H15 5340 6448 −2851 23 1

H16 7095 5694 −944 22 1

H17A 4442 8015 −1910 26 1

H17B 4538 7621 −3367 26 1

H11A 9150(40) 5840(15) 3287(19) 31(8) 1

H11B 8680(40) 5393(8) 1930(40) 35(8) 1

H12A 2140(30) 7424(11) −2300(20) 22(7) 1

H12B 1800(30) 7585(11) −3960(20) 36(8) 1

H12C 2380(30) 6952(7) −3320(30) 22(7) 1

H51A 2240(30) 6025(12) 5300(30) 25(7) 1

H51B 1910(40) 5741(10) 6460(30) 34(8) 1

|  |
| --- |

**Table 6.** Torsion angles [°].

|  |
| --- |

O7−B2−O1−B1 −169.86(18)

O2−B2−O1−B1 10.0(3)

O3−B1−O1−B2 −9.6(3)

O4−B1−O1−B2 −130.38(19)

O6−B1−O1−B2 110.4(2)

O1−B2−O2−B3 −2.7(3)

O7−B2−O2−B3 177.20(19)

O3−B3−O2−B2 −4.1(3)

O8−B3−O2−B2 177.55(19)

O8−B3−O3−B1 −177.99(18)

O2−B3−O3−B1 3.7(3)

O4−B1−O3−B3 122.9(2)

O6−B1−O3−B3 −115.3(2)

O1−B1−O3−B3 2.9(3)

O9−B4−O4−B1 −165.92(18)

O5−B4−O4−B1 14.9(3)

O3−B1−O4−B4 108.1(2)

O6−B1−O4−B4 −13.7(3)

O1−B1−O4−B4 −130.90(19)

O6−B5−O5−B4 −5.4(3)

O10−B5−O5−B4 172.80(17)

O9−B4−O5−B5 176.25(19)

O4−B4−O5−B5 −4.6(3)

O10−B5−O6−B1 −172.60(18)

O5−B5−O6−B1 5.5(3)

O3−B1−O6−B5 −118.0(2)

O4−B1−O6−B5 3.7(3)

O1−B1−O6−B5 121.92(19)

O17−B12−O11−B11 176.40(19)

O12−B12−O11−B11 −2.2(3)

O14−B11−O11−B12 118.39(19)

O13−B11−O11−B12 −3.4(3)

O16−B11−O11−B12 −122.48(19)

O11−B12−O12−B13 3.5(3)

O17−B12−O12−B13 −175.19(18)

O18−B13−O12−B12 −179.79(18)

O13−B13−O12−B12 1.4(3)

O18−B13−O13−B11 173.41(18)

O12−B13−O13−B11 −7.7(3)

O14−B11−O13−B13 −112.5(2)

O16−B11−O13−B13 125.42(19)

O11−B11−O13−B13 8.2(3)

O19−B14−O14−B11 −157.60(19)

O15−B14−O14−B11 20.6(3)

O13−B11−O14−B14 −148.52(18)

O16−B11−O14−B14 −26.9(3)

O11−B11−O14−B14 89.8(2)

O20−B15−O15−B14 179.92(18)

O16−B15−O15−B14 −1.5(3)

O19−B14−O15−B15 173.45(18)

O14−B14−O15−B15 −4.9(3)

O20−B15−O16−B11 171.03(18)

O15−B15−O16−B11 −7.5(3)

O14−B11−O16−B15 20.3(2)

O13−B11−O16−B15 142.47(18)

O11−B11−O16−B15 −97.9(2)

C6−C1−C2−C3 −0.7(3)

N1−C1−C2−C3 178.33(19)

C1−C2−C3−C4 1.5(3)

C2−C3−C4−C5 −0.7(3)

C2−C3−C4−C7 179.9(2)

C3−C4−C5−C6 −0.8(3)

C7−C4−C5−C6 178.60(18)

C2−C1−C6−C5 −0.7(3)

N1−C1−C6−C5 −179.83(18)

C4−C5−C6−C1 1.5(3)

C5−C4−C7−N2 121.8(2)

C3−C4−C7−N2 −58.8(3)

C16−C11−C12−C13 0.4(3)

N11−C11−C12−C13 175.10(19)

C11−C12−C13−C14 −1.5(3)

C12−C13−C14−C15 0.8(3)

C12−C13−C14−C17 179.3(2)

C13−C14−C15−C16 1.0(3)

C17−C14−C15−C16 −177.5(2)

C14−C15−C16−C11 −2.1(3)

C12−C11−C16−C15 1.4(3)

N11−C11−C16−C15 −173.44(19)

C13−C14−C17−N12 −102.6(2)

C15−C14−C17−N12 75.9(3)

|  |
| --- |

Symmetry transformations used to generate equivalent atoms:

|  |
| --- |

**Table 7.** Hydrogen bonds [Å and °].

*D*−H···*A* *d*(*D*−H) *d*(H···*A*) *d*(*D*···*A*) ∠(*D*H*A*)

O7−H7...O16 0.84 1.88 2.7206(19) 173.1

O8−H8...N11^i^ 0.84 2.01 2.820(3) 161.7

O9−H9...O7^ii^ 0.84 1.87 2.707(2) 173.4

O10−H10...O11^i^ 0.84 1.87 2.696(2) 169.3

O17−H17...O6^iii^ 0.84 1.85 2.686(2) 171.9

O18−H18...O20^iv^ 0.84 1.89 2.720(2) 171.7

O19−H19...N1^v^ 0.84 1.93 2.769(3) 176.0

O20−H20...O1 0.84 1.79 2.626(2) 172.1

N1−H1A...O3^iv^ 0.873(14) 2.37(3) 2.924(2) 122(3)

N1−H1B...O4^iv^ 0.885(14) 2.32(2) 3.094(2) 145(2)

N2−H2A...O13^vi^ 0.890(13) 2.058(16) 2.901(2) 158(2)

N2−H2B...O8 0.894(13) 2.62(2) 3.211(2) 124.1(19)

N2−H2B...O19^vii^ 0.894(13) 2.249(18) 2.988(2) 139.8(19)

N2−H2C...O10^viii^ 0.895(13) 2.072(17) 2.886(2) 151(2)

N11−H11A...O51^iii^ 0.875(14) 2.49(2) 3.171(3) 135(2)

N11−H11B...O18^ix^ 0.889(14) 2.33(2) 3.069(2) 141(3)

N12−H12A...O4 0.884(13) 2.014(15) 2.870(2) 163(2)

N12−H12B...O17^ii^ 0.893(13) 2.001(15) 2.868(2) 163(3)

N12−H12C...O51^x^ 0.904(13) 1.910(14) 2.806(2) 171(2)

O51−H51A...O8 0.861(13) 2.003(16) 2.801(2) 154(2)

O51−H51B...O14^vi^ 0.866(13) 1.945(15) 2.801(2) 170(3)

|  |
| --- |

Symmetry transformations used to generate equivalent atoms:

(i) x−1,y,z (ii) x−1,y,z−1 (iii) x+1,y,z (iv) x+1,y,z+1

(v) −x+2,y+1/2,−z+1 (vi) −x+1,y−1/2,−z+1 (vii) −x+1,y−1/2,−z

(viii) −x,y−1/2,−z (xi) −x+2,y−1/2,−z+1 (x) x,y,z−1

|  |
| --- |


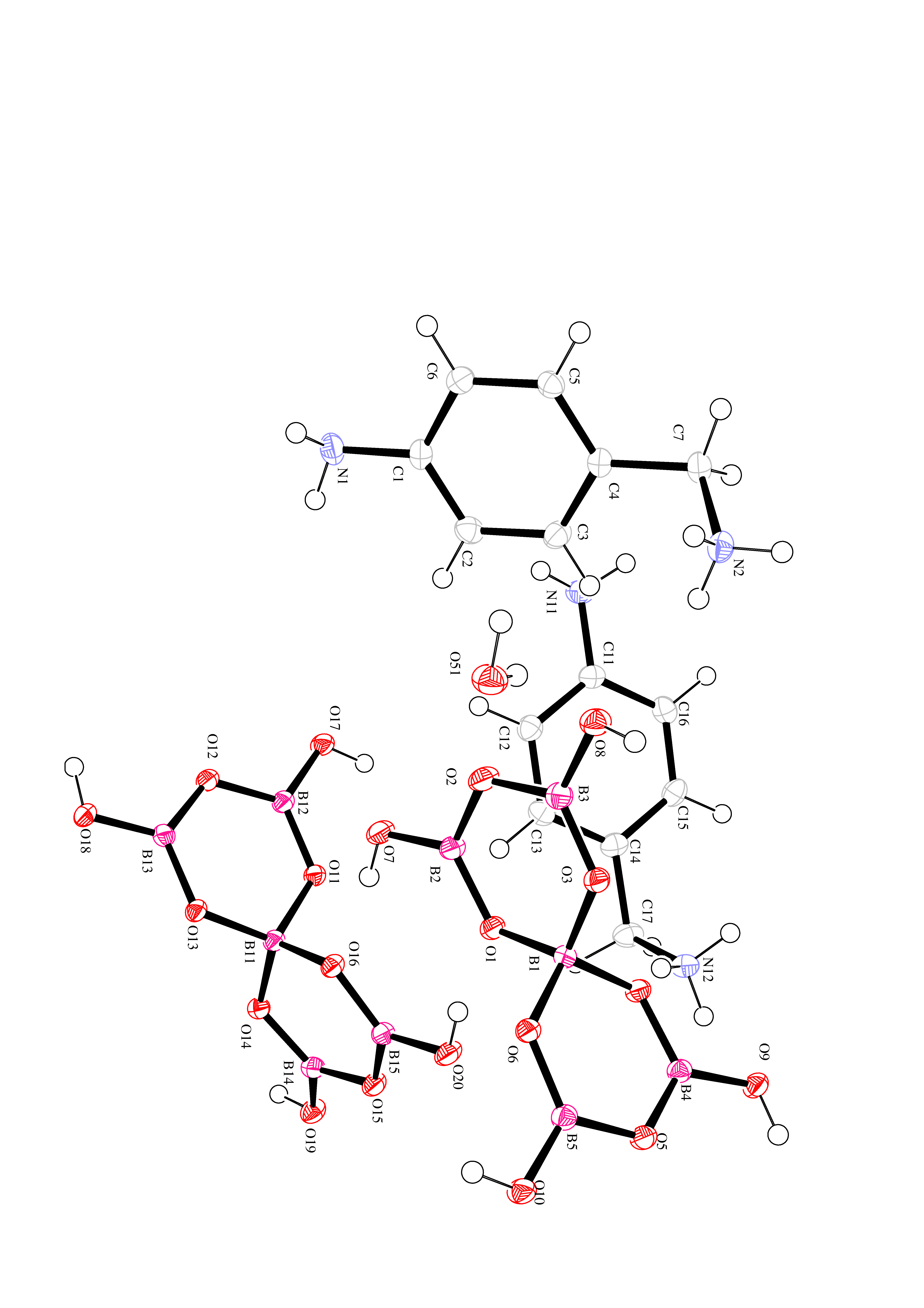

Supplement: Supplementary file 1 — Supplementary material 1 (DOCX 259 kb) [file 10876_2017_1205_MOESM1_ESM.docx]
