# Supplementary material for: Synthesis, XRD Studies and NLO Properties of [p-H2NC6H4CH2NH3][B5O6(OH)4]·1/2H2O and NLO Properties of Some Related Pentaborate(1−) Salts
Source: J Clust Sci. 2017 Apr 1;28(4):2087–95. doi: 10.1007/s10876-017-1205-1 (PMC7098061; doi:10.1007/s10876-017-1205-1)
Supplement: Supplementary file 2 — Supplementary material 2 (PDF 1380 kb) [file 10876_2017_1205_MOESM2_ESM.pdf]

Supplementary information for compound **1**

NMR spectra (^{1}H, ^{13}C and ^{11}B of both crude and recrystallized samples).

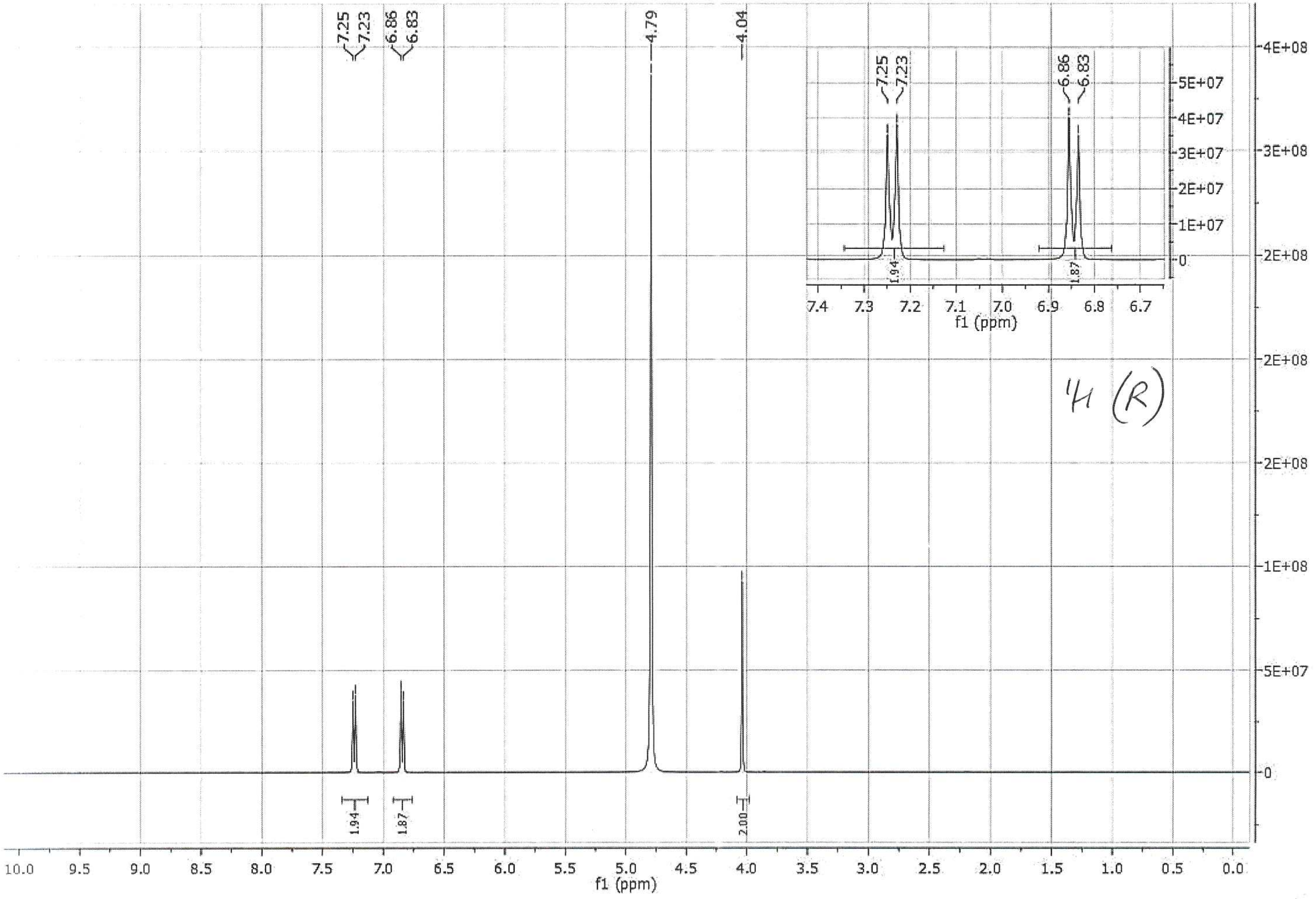

7.25
7.23
6.86
6.83
4.79
4.04
1.94
1.87
2.00
f1 (ppm)
¹H (R)

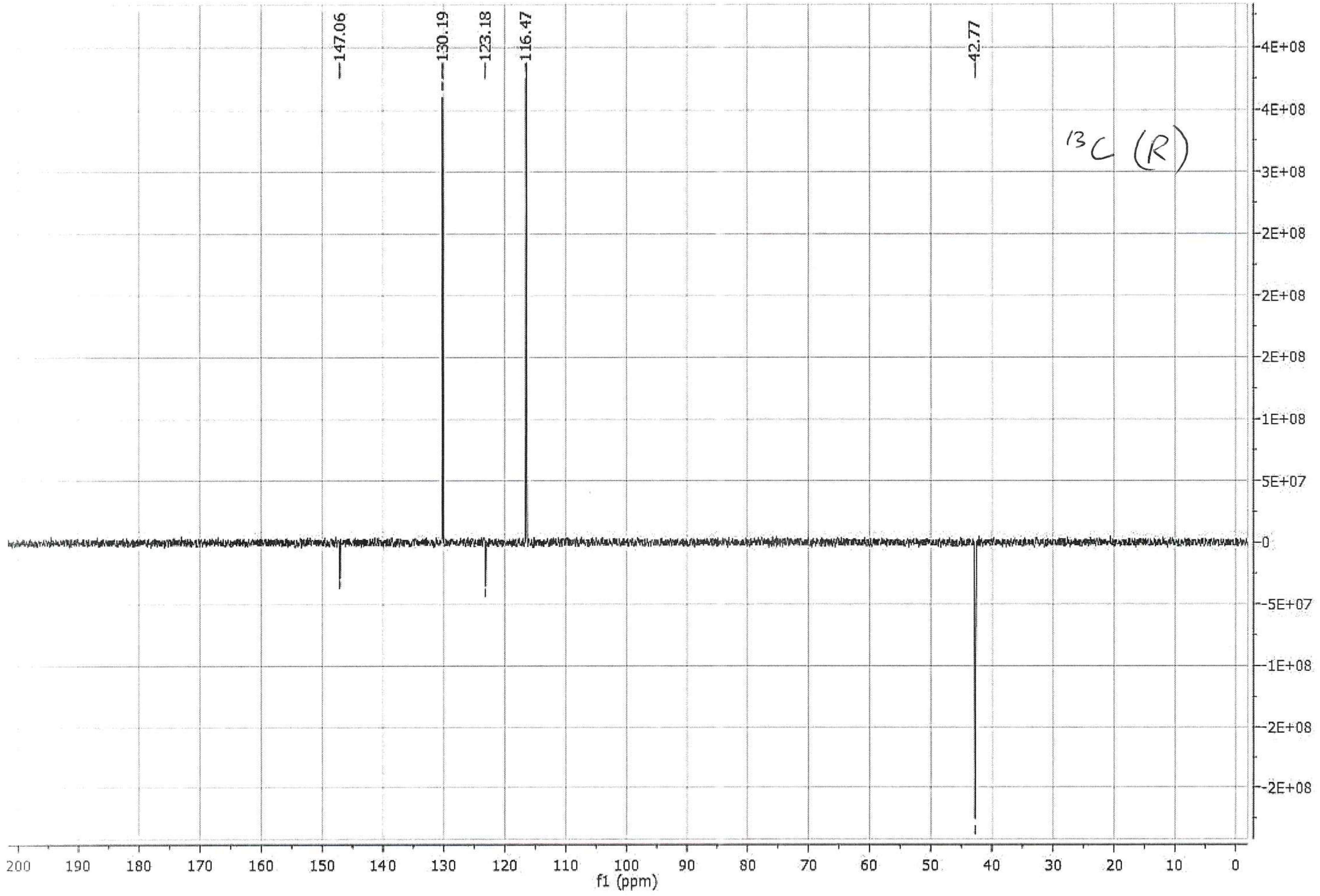
147.06
130.19
123.18
116.47
42.77
13C (R)
4E+08
4E+08
3E+08
2E+08
2E+08
2E+08
1E+08
5E+07
0
-5E+07
-1E+08
-2E+08
-2E+08
200 190 180 170 160 150 140 130 120 110 100 90 80 70 60 50 40 30 20 10 0
f1 (ppm)

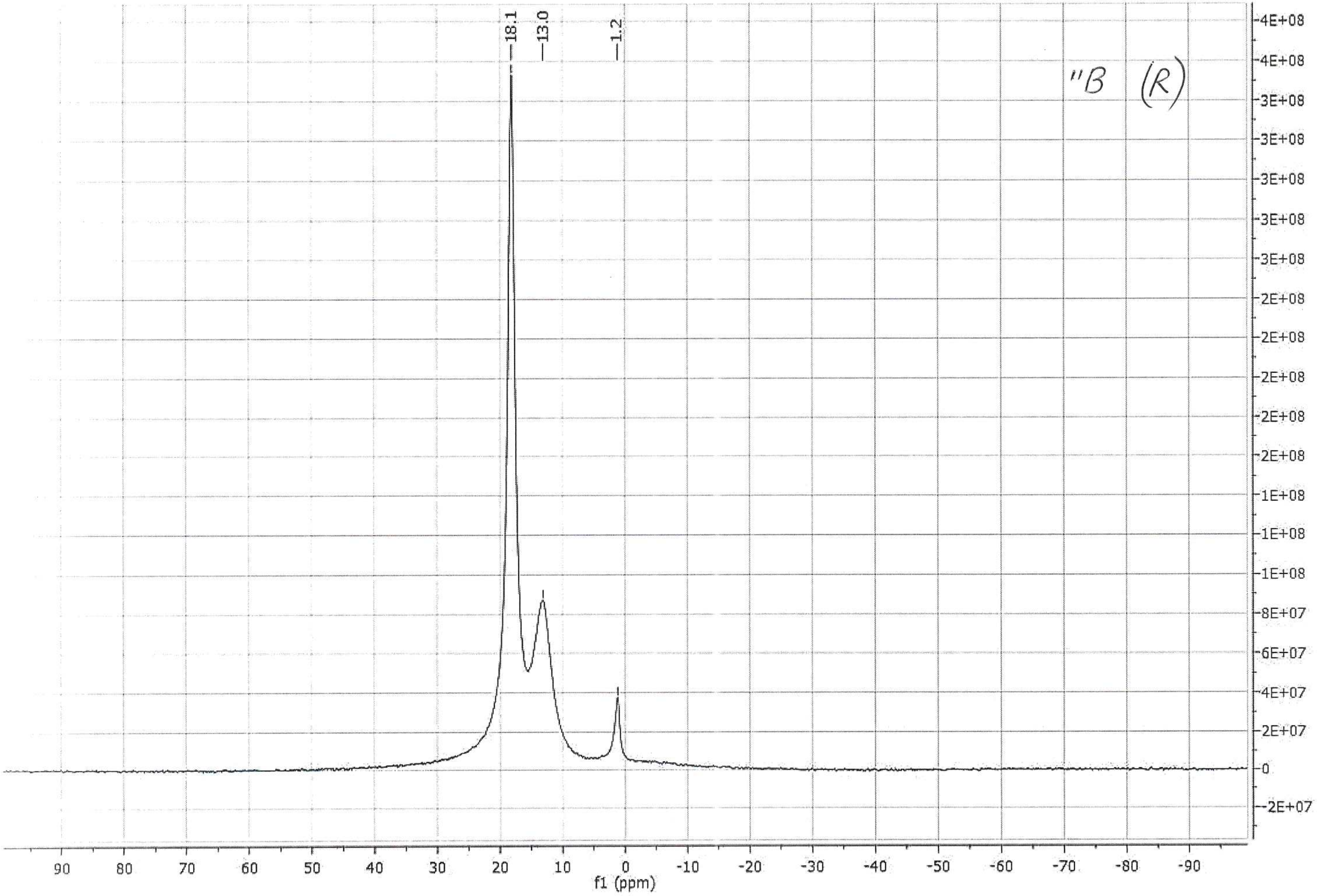
18.1
13.0
1.2
''B (R)
f1 (ppm)
90
80
70
60
50
40
30
20
10
0
-10
-20
-30
-40
-50
-60
-70
-80
-90
4E+08
4E+08
3E+08
3E+08
3E+08
3E+08
3E+08
2E+08
2E+08
2E+08
2E+08
2E+08
1E+08
1E+08
1E+08
8E+07
6E+07
4E+07
2E+07
0
-2E+07

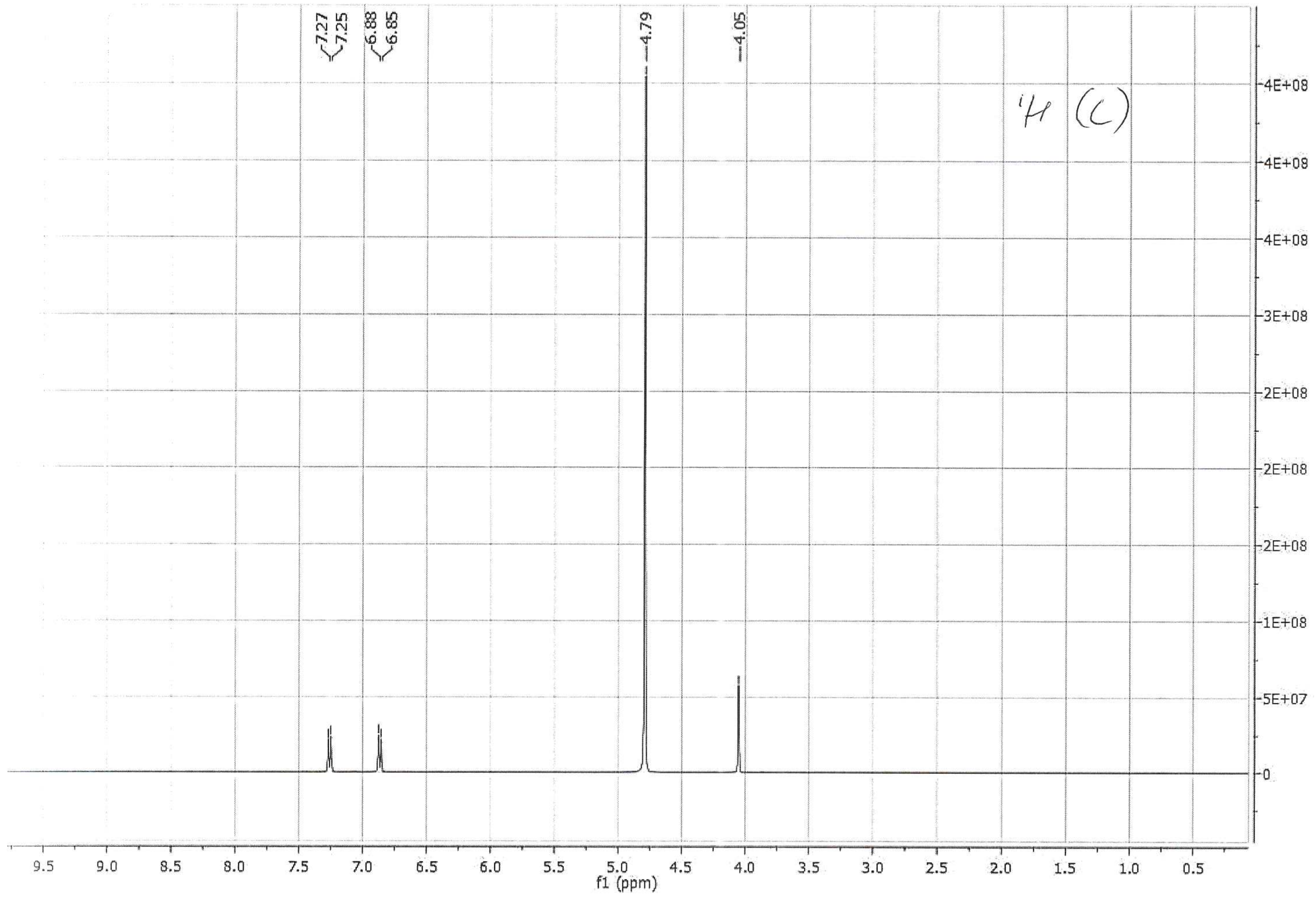

7.27
7.25
6.88
6.85
4.79
4.05
¹H (C)
4E+08
4E+08
4E+08
3E+08
2E+08
2E+08
2E+08
1E+08
5E+07
0
9.5
9.0
8.5
8.0
7.5
7.0
6.5
6.0
5.5
5.0
4.5
4.0
3.5
3.0
2.5
2.0
1.5
1.0
0.5
f1 (ppm)

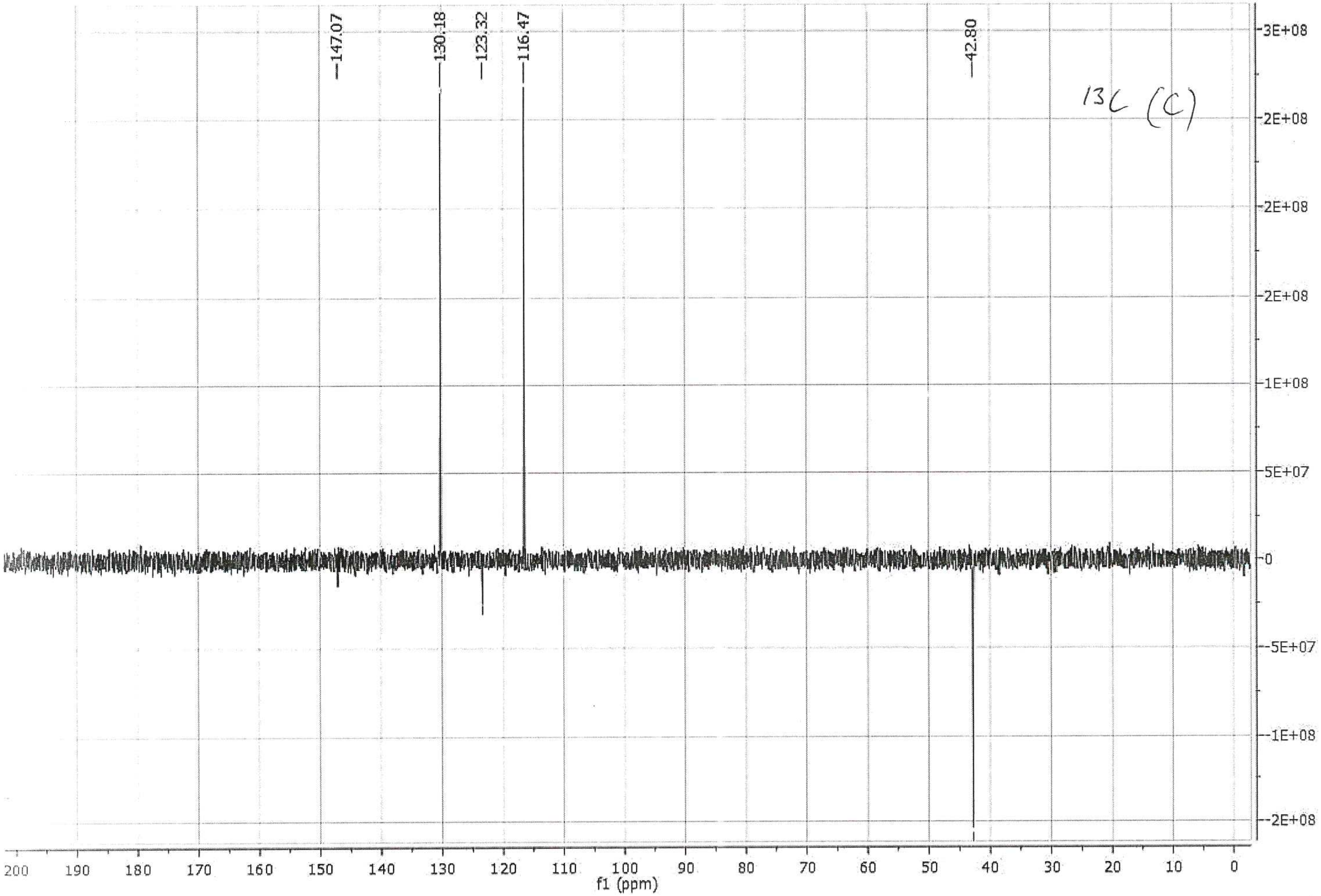
147.07
130.18
123.32
116.47
42.80
13C (C)
3E+08
2E+08
2E+08
2E+08
1E+08
5E+07
0
-5E+07
-1E+08
-2E+08
200
190
180
170
160
150
140
130
120
110
100
90
80
70
60
50
40
30
20
10
0
f1 (ppm)

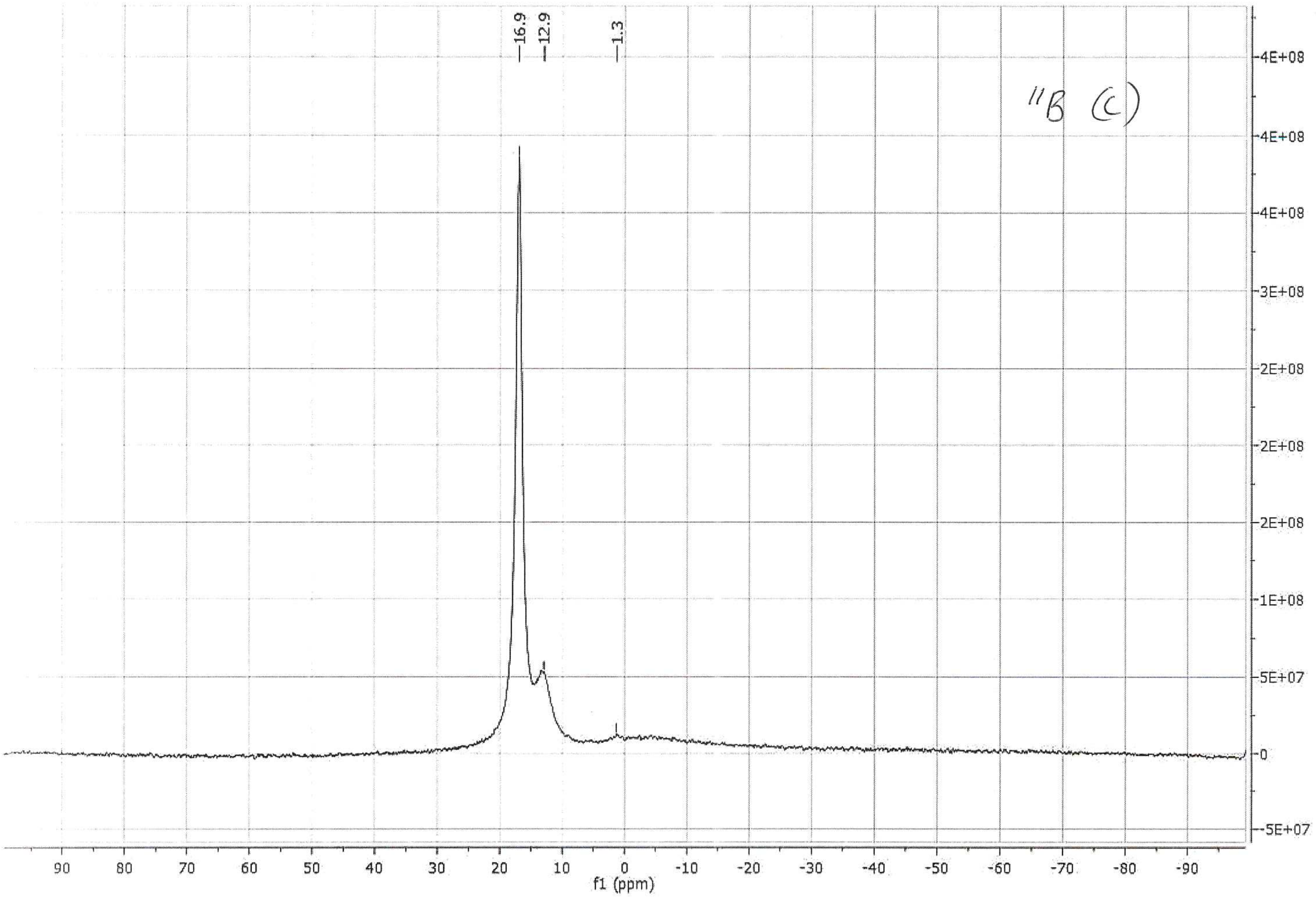
16.9
12.9
1.3
11B (C)
4E+08
4E+08
4E+08
3E+08
2E+08
2E+08
2E+08
1E+08
5E+07
0
-5E+07
90
80
70
60
50
40
30
20
10
0
-10
-20
-30
-40
-50
-60
-70
-80
-90
f1 (ppm)
