# Supplementary material for: Synthesis, XRD Studies and NLO Properties of [p-H2NC6H4CH2NH3][B5O6(OH)4]·1/2H2O and NLO Properties of Some Related Pentaborate(1−) Salts
Source: J Clust Sci. 2017 Apr 1;28(4):2087–95. doi: 10.1007/s10876-017-1205-1 (PMC7098061; doi:10.1007/s10876-017-1205-1)
Supplement: Supplementary file 3 — Supplementary material 3 (PDF 790 kb) [file 10876_2017_1205_MOESM3_ESM.pdf]

NLO DATA

Research samples

Name	Formula	Crude product	Recrystallized product	Reference
	$[C_6H_{13}N_2][B_5O_6(OH)_4]$	BECKETT-BANGOR-1c	-	[1]
4-Aminobenzylammonium pentaborate	$[C_7H_{11}N_2][B_5O_6(OH)_4]$	BECKETT-BANGOR-2c	BECKETT-BANGOR-2r	
Ammonium pentaborate	$[NH_4][B_5O_6(OH)_4]$	BECKETT-BANGOR-3c	BECKETT-BANGOR-3r	[2]
2-Amino-2-methyl-1-propanol pentaborate	$[NH_3CMe_2(CH_2OH)][B_5O_6(OH)_4]$	BECKETT-BANGOR-4c	BECKETT-BANGOR-4r	[3]
2-Amino-2-methyl-1,3-propanediol pentaborate	$[NH_3CMe(CH_2OH)_2][B_5O_6(OH)_4]$	BECKETT-BANGOR-5c	BECKETT-BANGOR-5r	[3]
1-(2-Hydroxyethyl)piperidinium pentaborate	$[(CH_2)_5NH(CH_2CH_2OH)][B_5O_6(OH)_4]$	BECKETT-BANGOR-6c	BECKETT-BANGOR-6r	[3]
1,2,3-Trimethylimidazolium pentaborate	$[1,2,3\text{-}Me_3C_3H_2N_2][B_5O_6(OH)_4]$	BECKETT-BANGOR-7c	BECKETT-BANGOR-7r	[4]
Potassium dihydrogen phosphate	KH_2PO_4	-	BECKETT-BANGOR-8r	
(S)-(+)-2-Pyrrolidinemethanol pentaborate	$[2\text{-}(HOCH_2)C_4H_7NH_2][B_5O_6(OH)_4]$	BECKETT-BANGOR-9c	BECKETT-BANGOR-9r	[5]

Chemical Hazard Information & Packaging (CHIP2) Please tick all those that apply				
O Oxidising ☐	F+ Extremely flammable ☐	F Highly flammable ☐	N Dangerous to environment ☐	E Explosive ☐
T+ Very toxic ☐	T Toxic ☐	C Corrosive ☐	Xn Harmful ☐	Xi Irritant ☐
Any other Hazards NOT covered above				
Signature: M A Beckett		Date: 25/4/16		

References

[1] H.-X. Liu, Y.-X. Liang, X. Jiang, *J. Solid State Chem.* 181, **2008**, 3243-3247.

[2] P. Becker, P. Held, L. Bohatý, *Cryst. Res. Technol.* 35, **2000**, 1251-1262.

[3] M.A. Beckett, P.N. Horton, M.B. Hursthouse, D.A. Knox, J.L. Timmis, *Dalton Trans.* 39, **2010**, 3944-3951.

[4] M.A. Beckett, P.N. Horton, M.B. Hursthouse, J.L. Timmis, *Polyhedron* 77, **2014**, 96-102.

[5] M.A. Beckett, S.J. Coles, R.A. Davies, P.N. Horton, C.L. Jones, *Dalton Trans.* 44, **2015**, 7032-7040.

Number	Sample code	Values (in mV)
1	1c	0.0
2	2c	1.2
3	3c	3.1
4	4c	0.3
5	5c	3.5
6	6c	1.4
7	7c	0.0
8	9c	1.8
9	2r	0.8
10	3r	2.2
11	4r	1.3
12	5r	1.24
13	6r	0.8
14	7r	0.0
15	8r	16.0
16	9r	3.3

KDP =17 mV

Research samples

Name of the free base	Formula	Notation
1,4-Diazabicyclo[2.2.2]octane	$[C_6H_{13}N_2][B_5O_6(OH)_4]$	MAB – 1C D
4-Aminobenzylamine	$[C_7H_{11}N_2][B_5O_6(OH)_4]$	MAB – 4-Amino
Ammonia	$[NH_4][B_5O_6(OH)_4]$	MAB – 3C RT<br>MAB – 3R RT
2-Amino-2-methyl-1-propanol	$[NH_3CMe_2(CH_2OH)][B_5O_6(OH)_4]$	MAB – 4C<br>MAB – 4R
2-Amino-2-methyl-1,3-propanediol	$[NH_3CMe(CH_2OH)_2][B_5O_6(OH)_4]$	MAB – 5C
(*S*)-(+)-2-Pyrrolidinemethanol	$[2\text{-}(HOCH_2)C_4H_7NH_2][B_5O_6(OH)_4]$	MAB – 9C<br>MAB – 9R
(*R*)-(-)-2-Pyrrolidinemethanol	$[2\text{-}(HOCH_2)C_4H_7NH_2][B_5O_6(OH)_4]$	MAB – RP
rac-2-Pyrrolidinemethanol	$[2\text{-}(HOCH_2)C_4H_7NH_2][B_5O_6(OH)_4]$	MAB – racP
(*S*)-(+)-2-Amino-1-propanol	$[CH_3CH(NH_3)CH_2OH][B_5O_6(OH)_4]$	MAB – SA
(*R*)-(+)-2-Amino-1-propanol	$[CH_3CH(NH_3)CH_2OH][B_5O_6(OH)_4]$	MAB – RA
rac-2-Amino-1-propanol	$[CH_3CH(NH_3)CH_2OH][B_5O_6(OH)_4]$	MAB – racA

Chemical Hazard Information & Packaging (CHIP2) Please tick all those that apply				
O Oxidising [ ]	F+ Extremely flammable [ ]	F Highly flammable [ ]	N Dangerous to environment [ ]	E Explosiv e [ ]
T+ Very toxic [ ]	T Toxic [X]	C Corrosive [ ]	Xn Harmful [ ]	Xi Irritant [ ]
Any other Hazards NOT covered above	not known - research sample - assume toxic			
Signature:		Date:		

Sample code	I_{2W} (mV)
MAB 4C	0
MAB cacp	0
MAB RA	0
MAB 5C	1
MAB 9R	2
MAB 3CRT	1
MAB RP	0
MAB 9C	0
MAB 1CD	7
MAB caca	2
MAB 4R	0
MAB 3RRT	1
MAB 5A	0
MAB 4 amine1	0

Input energy = 1 mJ/pulse

KDP = 12 mV
