# Supplementary material for: Synthesis, XRD Studies and NLO Properties of [p-H2NC6H4CH2NH3][B5O6(OH)4]·1/2H2O and NLO Properties of Some Related Pentaborate(1−) Salts
Source: J Clust Sci. 2017 Apr 1;28(4):2087–95. doi: 10.1007/s10876-017-1205-1 (PMC7098061; doi:10.1007/s10876-017-1205-1)
Supplement: Supplementary file 4 — Supplementary material 4 (PDF 118 kb) [file 10876_2017_1205_MOESM4_ESM.pdf]

Suplementary information TGA/DSC for **1**.

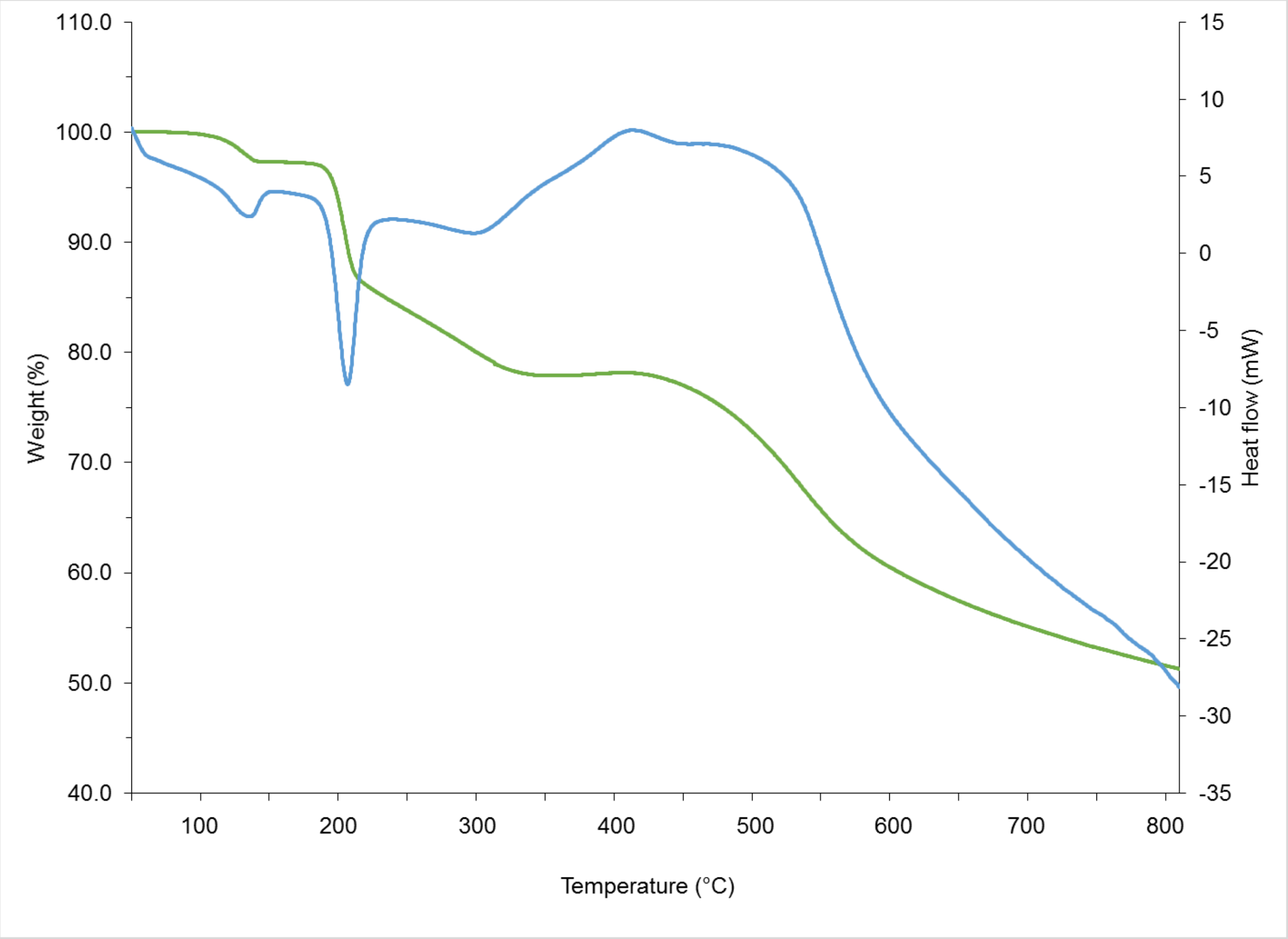

Thermal (TGA/DSC) plots (in air) for compound **1**. The first two low temperature weight loss steps are endothermic whereas the processes associated with the higher temperature later steps are exothermic.
